# Translation and Cross-Cultural Adaptation of the LYMPH-ICF Instrument for Lymphedema into Portuguese/Brazil

**DOI:** 10.1590/0034-7167-2023-0137

**Published:** 2024-06-14

**Authors:** Ana Paula Oliveira Santos, Samantha Karlla Lopes de Almeida Rizzi, Gil Facina, Afonso Celso Pinto Nazário, Simone Elias

**Affiliations:** I Universidade Federal de São Paulo. São Paulo, São Paulo, Brazil

**Keywords:** Breast Neoplasms, Lymphedema, International Classification of Functioning, Disabiliy and Health, Health Care, Patient Health Questionnaire, Neoplasias de la Mama, Linfedema, Classificación Internacional del Funcionamiento, de la Discapacidad y de la Salud, Traducción, Cuestionario de Salud del Pacient

## Abstract

**Objective:**

The aim of this study is to cross-culturally adapt the Lymphoedema Functioning, Disability and Health Questionnaire Lymphoedema (LYMPH-ICF) instrument into Brazilian Portuguese and conduct a pilot application (n = 10), without psychometric pretensions.

**Method:**

Methodological research was conducted, following the steps of translation, synthesis, back-translation, and evaluation by the expert committee. Two translators, two back-translators, and twelve professionals participated in the expert committee. A pretest was carried out with 10 patients with secondary lymphedema due to breast cancer. The degree of agreement was determined by the content validity coefficient.

**Results:**

It was necessary to modify 8 out of the 29 questions comprising the questionnaire, which exhibited idiomatic disagreement. However, despite these changes, there were no indications of impairments, as content reliability was achieved through a validity coefficient of 0.90.

**Final Considerations:**

The instrument was successfully translated and cross-culturally adapted for Brazil with a high level of agreement.

## RESUMEN

**Objetivo:**

Realizar la adaptación transcultural del instrumento Cuestionario de Funcionamiento, Discapacidad y Salud del Linfedema (LYMPH-ICF) al portugués de Brasil y una aplicación piloto (n = 10), sin pretensiones psicométricas.

**Método:**

Investigación metodológica, siguiendo los pasos de traducción, síntesis, retrotraducción y evaluación por parte del comité de expertos. Participaron dos traductores, dos retrotraductores y doce profesionales para el comité de expertos. Se realizó una prueba piloto con 10 pacientes con linfedema secundario al cáncer de mama. Y el grado de concordancia se obtuvo mediante el coeficiente de validez de contenido.

**Resultados:**

Hubo necesidad de modificar 8 de las 29 preguntas que componen el cuestionario, las cuales mostraron discordancia idiomática. Sin embargo, a pesar de que se realizaron dichas modificaciones, no hubo indicación de perjuicios, ya que se alcanzó la confiabilidad del contenido mediante la obtención de 0,90 en el coeficiente de validez.

**Conclusiones:**

El instrumento fue traducido y adaptado transculturalmente para Brasil con un alto grado de concordancia.

## INTRODUCTION

Breast cancer stands as one of the primary health concerns among women due to its elevated mortality and morbidity rates. Even with adjuvant chemotherapy, the survival rate for metastatic breast cancer remains below 30% over a five-year span. According to the International Agency for Research on Cancer (IARC) in 2020, female breast cancer has surpassed lung cancer as the leading cause of global cancer incidence, with an estimated 2.3 million new cases, comprising 11.7% of all cancer cases^(1, 2)^.

In Brazil, estimates for each year within the 2023-2025 triennium project 704 thousand new cancer cases (excluding non-melanoma skin cancer cases, totaling 483 thousand). Breast cancer emerges as the most prevalent, representing 20.3% of female tumor cases, in alignment with global patterns^(3)^.

Advancements in treatment offer a spectrum of therapies, including surgery, radiotherapy, chemotherapy, hormone therapy, and targeted biological therapy, tailored to disease stage and tumor type. Surgical axillary dissection and/or radiotherapy in the lymph node region, however, give rise to a significant comorbidity, lymphedema^(4, 5, 6)^.

Lack of standardization in lymphedema assessment results in considerable variation in its incidence post-breast cancer surgery. Rates range from approximately 9 to 40%, with mastectomy-associated rates of 24 to 49%, lumpectomy with axillary dissection ranging from 4 to 28%, and surgery with radiotherapy from 5 to 34%^(7)^.

Lymphedema arises from lymphatic flow obstruction or absence, characterized by extracellular accumulation of water, plasma proteins, extravascular blood cells, and cellular products due to deficient lymphatic transport. It may manifest alongside complications such as cellulitis, erysipelas, lymphangitis, and, rarely, lymphangiosarcoma^(7,8,9,10,11)^. While not inherently life-threatening, these changes induce various disorders in the affected limb, impacting aesthetics, psychosocial well-being, and functionality^(12, 13, 14)^.

Functionality serves as the third-largest health indicator, aiding therapeutic guidance by pinpointing environmental factors as facilitators or barriers in function and action performance^(15, 16, 17, 18)^. Although breast cancer significantly influences functionality, functional indicators receive less scrutiny than survival and mortality indicators. Implementing functionality assessment represents a global trend in outcome integration, crucial in the evaluation of cancer patients, with or without lymphedema, given their documented impairments across physical, social, and emotional spheres^(19, 20, 31, 22)^. Notably, negative implications of barriers to early treatment access may further reduce functionality in women with breast cancer^(23)^.

Given the scarcity of translated and validated instruments in Portuguese capable of evaluating functionality related to lymphedema, specifically post-axillary surgery for breast cancer treatment, we elected to translate and cross-culturally adapt the Lymphoedema Functioning, Disability, and Health Questionnaire Lymphoedema (LYMPH-ICF) questionnaire, previously validated in other languages.

The utilization of non-specific scales and questionnaires for upper limb lymphedema undermines the methodological quality of studies by failing to offer pertinent data regarding the experiences of this demographic. Furthermore, formulated on the foundation of the International Classification of Functioning, Disability, and Health (ICF), the tool encompasses various dimensions associated with physical, mental, domestic, social, and mobility concerns, rendering it a crucial assessment instrument in clinical settings for individuals managing lymphedema. It facilitates comprehension of functional limitations, constraints in daily activities, and limitations on social engagement.

## OBJECTIVE

To translate and cross-culturally adapt the “Lymphoedema Functioning, Disability and Health Questionnaire Lymphoedema - LYMPH-ICF” instrument into Brazilian Portuguese.

## METHODS

### Ethical considerations

The study design adhered to the guidelines of Resolution 466/2012 of the National Health Council and was approved by the Research and Ethics Committee of the Federal University of São Paulo (UNIFESP), Certificate of Presentation for Ethical Appreciation - CAAE No. 35272614.0.0000.5274, Opinion No. 2,198,617. Initially, Dr. Nele Devoogdt, the first author of the original and English-translated versions, was electronically requested authorization to conduct this study. All participants provided signed Informed Consent Forms.

### Study type

This is a methodological study of translating and cross-culturally adapting the “Lymphoedema Functioning, Disability and Health Questionnaire Lymphoedema - LYMPH-ICF” instrument into Brazilian Portuguese. The LYMPH ICF questionnaire, based on the International Classification of Functioning (ICF) terminology, evaluates impairments related to functionality, activity limitation, and participation restriction in patients post-axillary surgery due to breast cancer. Comprising 29 questions, it was developed based on information from patients with secondary lymphedema due to breast cancer.

Patients score responses using an 11-point numerical scale (0 to 10). For each question, patients indicate the number that best corresponds to their situation. “0” is assigned if the patient has no problems related to their complaint, and “10” if they experience very serious issues. The option “does not apply” indicates that the complaint does not relate to the patient. Questions are grouped into 5 categories: 1) physical issues: pain, skin sensation, and movement (7 questions); 2) mental function (4 questions); 3) domestic activities (4 questions); 4) mobility activities (8 questions), and 5) social and life activities (6 questions). A higher score indicates a greater impact of lymphedema on the functionality of breast cancer patients.

Domain scores range from 0 to 100, calculated by summing the scores of the 29 questions, dividing by the total number of responses, and multiplying by 10. The author of the original version emphasizes, regarding function impairments, that according to the World Health Organization (WHO) taxonomy, activity limitations and participation restrictions are classified as: 0 to 4% (no problems), 5 to 24% (mild problem), 25 to 49% (moderate problem), 50 to 95% (severe problem), and 96 to 100% (very severe problem)^(24)^.

### Methodological Procedure

**Figure 1:**
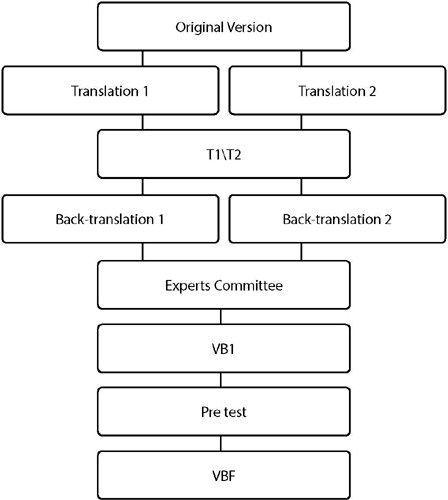
Stages of translation and cross-cultural adaptation

Note: T1/T2: synthesis of translations by the authors; VB1: First Brazilian Version; VBF: Final Brazilian Version

To adapt the LYMPH-ICF, we followed the recommendations of the BEATON Guideline^(25)^, aiming to ensure semantic, idiomatic, cultural, and conceptual equivalence between the original instrument and the adapted version. Thus, the five necessary steps for Translation and Cultural Adaptation (TCA) of an instrument were conducted, including translation, synthesis of translations, back-translation, evaluation by the expert committee, and pre-testing, as illustrated in Figure 1.

### Study Setting and Data Collection

The study setting and data collection process varied at each stage of the research. In the initial phase, two independent Brazilian translators, experienced in oncology and lymphology and familiar with the research objective, conducted the initial translation from English to Portuguese, identified as T1 and T2. Following this step, the translations (T1 and T2) were consolidated by the responsible researchers into a unified version translated into Portuguese (T1/2).

During the back-translation phase, the consolidated version (T1/2) was provided to two native English-speaking translators for back-translation into the instrument’s original language (BT1 and BT2). These translators were unaware of the research objectives. The instrument versions (original, synthesis of translations into Portuguese, and back-translation) underwent semantic and content validation through a committee comprising 12 specialists from various Brazilian states (São Paulo, Rio de Janeiro, Mato Grosso, Rio Grande do Sul, Brasília, Bahia, and Santa Catarina). These specialists were selected based on their Brazilian nationality, English proficiency, and expertise in lymphology, oncology, and/or mastology. Invitations and procedural instructions were electronically sent to the selected specialists.

Regarding translation and cross-cultural adaptation, the literature presents significant divergence regarding the required number of participants for forming the expert committee. Despite identifying approximately 30 guidelines, no consensus indicates a single standard. Therefore, specialists were selected based on numbers reported in other studies as referenced^(26)^.

After comparing the versions with the original text, potential flaws in 8 questions were identified and analyzed concerning discordant points. By minimizing possible linguistic, cultural, and comprehension biases, the construction of the first Brazilian version of the instrument (VB1) was facilitated.

Subsequently, a pre-test was conducted with VB1 to determine the adequacy of procedures and identify any remaining incomprehensible items. This stage enabled the identification and correction of potential errors before the research commenced. The pre-test involved 10 women with lymphedema, following the published version of the Translation and Cultural Adaptation (TCA) regarding the required number of participants for questionnaire development for lower limb lymphedema. The recruited patients were from the Physical Therapy Outpatient Clinic of the Mastology Discipline, Department of Gynecology, Federal University of São Paulo (UNIFESP). They had upper limb lymphedema secondary to breast cancer, of any grade, and possessed at least a completed primary school education level. Illiterate patients and those with visual or cognitive impairments that could hinder questionnaire reading and comprehension were excluded.

The study also collected demographic variables (gender, age, marital status) and clinical variables (date of lymphedema diagnosis and grade) to characterize the study population.

Following the pre-test, the process of elaborating the final version commenced, seeking consensus among the expert committee regarding semantics (ensuring transfer of meaning from concepts in the original instrument to the new version), idiomatic equivalence (accurate translation of language expressions and colloquialisms), cultural factors (capturing daily life experiences in questionnaire items), and conceptual aspects (analyzing concepts and word meanings in English and Brazilian cultural contexts)^(27, 28)^.

### Data Analysis

Following the completion of the pre-test, the questions underwent another round of evaluation by the expert committee to gauge the level of semantic, idiomatic, cultural, and conceptual agreement using the Content Validity Coefficient (CVC). The evaluation of questions employs a 4-point Likert scale, where 1 signifies “not clear”; 2 “somewhat unclear”; 3 “quite clear”; and 4 “very clear”. The resulting score was calculated by summing up the ratings for items 3 and 4 and dividing by the total number of responses. A score above 0.80 is considered satisfactory to ensure agreement among experts.

## RESULTS

Following the initial procedures (translation and back-translation) detailed in Chart 1, to appropriately adapt the LYMPH ICF into Brazilian Portuguese, the expert committee conducted an evaluation. They proposed minor modifications to the T1/2 version to enhance participant comprehension, such as incorporating more common terms from the everyday language of the Brazilian population. For instance, the phrase “how well can you?” was replaced with “can you?” in the statement.

**Chart 1:** Original version questions, translation, and back-translations of LYMPH-ICF questions

Statement	Original	Translation T1/T2	Back-translation 1	Back-translation 2
**Pain, skin sensations, functions, and movement**				
	Does your arm:	*Em relação ao seu braço:*	Does it arm:	Does it arm:
1	Feel heavy?	*Sente pesado?*	Feel heavy	Feel heavy?
2	feel stiff?	*Sente rígido?*	Feel stiff?	Does it feel tight?
3	Feel swollen?	*Sente inchado?*	Feel swollen?	Does it feel swollen?
4	Feel like it has lost strength	*Sente que perdeu a força?*	Feel as if you have lost your arm’s strength?	Do you feel like it has lose strength?
5	Tingle?	*Formigamento?*	Tingling?	Does it tingle?
6	Hurt?	*Dor?*	Any pain?	Does it hurt?
7	Have a tense skin?	*Tem a pele tensa?*	Have tense skin?	Is the skin tight?
**Mental Function**				
8	Due to you arm problems Do you feel sad?	*Devido ao problema no seu braço você se sente triste?*	Due to problems with your arm:Do you feel sad?	Do you feel sad?
9	Do you feel discouraged?	*Você se sente desencorajado?*	Do you feel discouraged?	Do you feel discouraged?
10	Do you have a lack of self-confidence?	*Você se sente falta de auto-confiança?*	Do you feel a lack of self-confidence?	Do you feel a lack of self-confidence?
11	Do you feel stressed?	*Você se sente estressado?*	Do you feel stressed?	Do you feel stressed?
**Domestic Activities**				
	How well are you able to	*Quão bem você é capaz*	How well are you able to	How well are you able to
12	Clean? (scrub, vaccum, mop).	*Limpar? (esfregar, aspirar, passar pano)*	Cleaning? (rubbing, use a vacuum cleaner, wipe with a cloth)?	Clean?(scrubbing, vacuuming, dusting)?
13	Cook?	*Cozinhar?*	Cook?	Cooking?
14	Iron?	*Passar ferro?*	Iron clothes?	Ironing?
15	Work in the garden?	*Trabalhar no jardim?*	Work in the garden?	Work in the garden?
**Mobility Activities**				
	How well are you able to	*Quão bem você é capaz de*	How well are you capable of	How well are you able to
16	Perform tasks with the arm elevated (eg, hang out the laundry)	*Executar tarefas com o braço elevado (por exemplo, estender a roupa)*	Performing tasks with a raised arm (such as putting clothes on the line)?	Perform tasks with your arm elevated (for instance, hanging clothes)?
17	Lift or carry heavy objects (eg, leg a filled bucket or shopping bags)?	*Levantar ou carregar objetos pesados (por exemplo, um balde cheio ou sacola de compras)?*	Lift or carry heavy objects (such as a bucketful of water or a full shopping bag)?	Lift or carry heavy objects (for instance, a filled bucket or a grocery bag)?
**Mobility Activities**				
18	Sleep on the affected side?	*Dormir sobre o lado afetado?*	Sleeping on the affected side?	Sleep over the affected side?
19	Perform computer work?	*Fazer trabalho no computador?*	Using a computer?	Work on your computer?
20	Sunbathe	*Tomar sol?*	Sitting out in the sun?	Sunbathing?
21	Drive a car?	*Dirigir um carro?*	Driving a car?	Drive a car?
22	Walk (>2 km)?	*Andar (>2 km)?*	Walking (>2 km)?	Walk (>2 km)?
23	Ride a bike?	*Andar de bicicleta?*	Riding a bike?	Ride a bike?
**Social and Life Activities**				
	How well are you able to	*Quão bem você é capaz de*	How well are you capable of	How well are you able
24	Go on vacation?	*Ir de férias?*	Going on a holiday?	Take a vacation?
25	Perform your hobbies?	*Realizar seus passatempos?*	Dedicating yourself to you hobbies?	Do your hobbies?
26	Practice sports?	*Praticar esportes?*	Engaging in sports activities?	Practice sports?
27	Wear your clothes of choice	*Usar suas roupas escolhidas?*	Using a given item of clothing?	Use chosen clothes?
28	Do your job?	*Fazer seu trabalho?*	Performing at your job?	Perform your job?
29	Do social activities (eg, going to parties, concerts, restaurant)?	*Fazer suas atividades sociais (por exemplo, ir a festas, concertos e restaurantes)?*	Conduct social activities (i.e.: go to parties, concerts or restaurants)?	Perform your social activities (for instance, go to parties, concerts, restaurants)?

*Legend: T1: translated version 1, T2: translated version 2.*

Additionally, subtle adjustments were made to the 8 questions exhibiting idiomatic and conceptual discrepancies, as illustrated in Chart 2, resulting in the creation of the first Brazilian version (VB1).

**Chart 2:** Changes made after evaluation by the expert committee and consensus among the lead researchers

Question	T1/T2	VB1
**Statement**	*Quão bem você é capaz?*	*Você consegue?*
2	*Rígido?*	*Endurecido?*
6	*Dor?*	*Dor no braço?*
7	*A pele tensa?*	*A pele esticada?*
9	*Desencorajada?*	*Desanimada?*
16	*Passar ferro?*	*Passar roupa?*
19	*Fazer trabalho no computador?*	*Trabalhar no computador?*
24	*Ir de férias?*	*Sair de férias?*
27	*Usar suas roupas escolhidas?*	*Usar as roupas que escolheu?*

*Legend: T1/T2: Synthesis of translated versions, VB1: First Brazilian Version.*

In the VB1 pre-test, 10 women with an average age of 55.6 years (SD ± 13.65) were included, with 50% being white. All patients underwent axillary lymphadenectomy and radiotherapy for breast cancer treatment, resulting in secondary lymphedema due to oncological treatment. Lymphedema had been diagnosed on average for 20.6 months (SD ± 21.41). At least one patient responded “does not apply” to 8 out of the 29 questionnaire questions. It’s worth noting that the questions were marked as “does not apply” solely due to the patient’s routine, not because of comprehension difficulties or because it did not reflect the reality for the Brazilian population in general. The questions marked as “does not apply” were: question 14: “ironing”; question 15: “working in the garden”; question 18: “sleeping on the affected side”; question 19: “working on the computer”; question 21: “driving a car”; question 22: “walking >2 km”; question 23: “riding a bicycle”; and question 26: “engaging in sports”.

**Figure 2:**
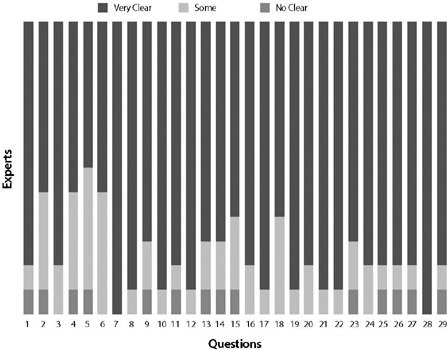
Agreement of the expert committee on the final Brazilian version

After evaluating the responses of the Expert Committee, which showed a degree of agreement by the IVC of 0.90 (Figure 2), no final changes to the translated instrument were necessary, and it was considered the Final Brazilian Version (VBF), suitable for application in the studied population.

Following the evaluation of the Expert Committee’s responses, no final changes to the translated instrument were necessary, and it was deemed the Final Brazilian Version (VBF), suitable for use in the studied population.

## DISCUSSION

Questionnaires for assessment are common tools in various healthcare fields, often used in populations different from those for which they were originally created. In these cases, cross-cultural adaptation is necessary in the preparation and application of questionnaires, as their validity depends not only on translation but also on contextualization to the new cultural setting to maintain their psychometric properties^(27, 28, 29)^.

The initial translations showed similar results, and the translators did not indicate difficulties because they are Brazilians with experience in the fields of lymphology and oncology, thus semantic deviations were minimized. Additionally, the translators’ independent work ensured that interpretative errors and peculiarities in writing style were avoided. The translations of the title and instructions from T1 and T2 of the LYMPH-ICF questionnaire were similar and did not require changes when transformed into T1/T2.

Following BEATON’s criteria (2000), which suggests that back-translation should be blind (back-translators should not have access to the original questionnaire) and they should be native in the original language of the document, fluent in the target language, and should not have technical knowledge about the subject ^(25)^. The synthesis of translations underwent back-translation, aimed at identifying words that were not clear in the original language and finding inconsistencies or conceptual errors in the final Brazilian version when compared to the original version^(30)^.

The need to make minor changes in the back-translation process was identified after evaluation by the expert committee, as the back-translators used different pronouns and prepositions, as well as synonymous words, resulting in different back-translations with possible disagreement regarding idiomatic equivalence in 8 questions. Although minor changes were made as suggested in some items, they did not alter the theoretical dimension of the instrument compared to the original.

The pre-test is a preliminary application of the instrument in a small sample that reflects the characteristics of the target population, in order to assess the suitability of items regarding their meaning and comprehension difficulty^(31)^. Due to the low education level of the population served in our service, this step required an explanation of how to score difficulties using the visual analog scale used in the questionnaire, but this does not undermine the applicability of the questionnaire in clinical practice, as the clarity of the theoretical language of the instrument’s questions was confirmed by the high level of agreement among experts, with a CVC value of 0.90 on the Likert scale, as recommended in the literature.

In a recent systematic review to evaluate the psychometric properties of questionnaires measuring the quality of life of patients with secondary lymphedema due to breast cancer, among the 9 instruments evaluated using the COSMIN Risk of Bias checklist, the LYMPH ICF was classified as having the best evidence of content validity regarding relevance, comprehensiveness, and understandability^(31)^.

In terms of feasibility, the LYMPH ICF features concise, lucid, and straightforward questions, accompanied by an 11-point numerical scale that is easily comprehensible to both patients and clinicians. The questionnaire also includes a simple scoring system and can be completed within 5 to 10 minutes^(31)^.

The procedures used to adapt and translate an instrument into a new language involve distinct steps that necessitate impartial, consistent, and methodologically compliant actions. This ensures that the values reflected by the instrument and the meanings of its items remain consistent across cultures, guaranteeing that its translation maintains the same applicability as the original^(32, 33)^.

The findings align with others reported in the scientific literature, supporting the feasibility of a Portuguese version. This is evidenced by the fact that the original instrument^(27)^ has been translated into various languages such as French, Danish, and Chinese using similar methods, alongside assessments of psychometric issues that ensure the reliability and validity of the LYMPH-ICF and its analogous version for lower limb lymphedema^(34, 35, 36, 37)^.

### Limitations of the Study

Despite adhering to rigorous methodological procedures, certain limitations must be acknowledged. Firstly, the pre-test sample size was relatively small, comprising only 10 women with secondary lymphedema resulting from breast cancer. A larger sample could yield a more comprehensive evaluation of the questionnaire’s comprehension and applicability, facilitating a stronger validation of the Brazilian version. Moreover, participant selection was confined to a single healthcare center, potentially limiting result generalization to other populations or medical contexts.

Another potential limitation pertains to the sample’s homogeneity in sociodemographic characteristics, such as age and education level. The majority of study participants had an average age of around 55 years and possessed at least a primary education. This homogeneity could impact result representativeness, particularly concerning patients from diverse age groups or educational backgrounds. A more diversified approach could offer further insights into the instrument’s understanding and acceptance across various population groups, enabling a more comprehensive and inclusive questionnaire adaptation for the Brazilian context.

### Contributions to Nursing, Health, or Public Policy

This study has provided a valuable instrument for the fields of nursing, health, and public policy by introducing a questionnaire on functionality, disability, and lymphedema health, translated and cross-culturally adapted into Brazilian Portuguese. This initiative empowers professionals to evaluate and monitor lymphedema presence and progression in women undergoing breast cancer treatment surgery. This marks a pivotal initial step in LYMPH-ICF validation, furnishing an essential tool for clinical practice and research in the field while significantly enhancing patient care.

## FINAL CONSIDERATIONS

Following translation and cross-cultural adaptation processes, the evaluation of semantics, idiomatic equivalences, and cultural and conceptual factors culminated in the development of a Brazilian Portuguese instrument version with a high degree of agreement (CVC 0.90). It is recommended, henceforth, to employ this instrument in future studies involving the Brazilian population afflicted by secondary lymphedema due to breast cancer, with a focus on psychometric analysis, statistical reliability, and instrument validation.

## Data Availability

https://doi.org/10.48331/scielodata.2IQUF1
