# Supplementary material for: Translation and Cross-Cultural Adaptation of the LYMPH-ICF Instrument for Lymphedema into Portuguese/Brazil
Source: Rev Bras Enferm. 2024 Jun 14;77(2):e20230137. doi: 10.1590/0034-7167-2023-0137 (PMC11178305; doi:10.1590/0034-7167-2023-0137)
Supplement: Translation

# Translation: Tradução e adaptação transcultural para o português/Brasil do instrumento LYMPH-ICF para linfedema

**DOI:** 10.1590/0034-7167-2023-0137pt

**Authors:** Ana Paula Oliveira Santos, Samantha Karlla Lopes de Almeida Rizzi, Gil Facina, Afonso Celso Pinto Nazário, Simone Elias

**Affiliations:** I Universidade Federal de São Paulo. São Paulo, São Paulo, Brasil

**Keywords:** Neoplasias da Mama, Linfedema, Classificação Internacional de Funcionalidade, Incapacidade e Saúde, Tradução, Questionário de Saúde do Paciente

## Abstract

**Objetivo:**

realizar a adaptação transcultural do instrumento *Lymphoedema Functioning, Disability and Health Questionnaire Lymphoedema* (LYMPH-ICF) para o português do Brasil e uma aplicação piloto (n = 10), sem pretensão psicométrica.

**Método:**

pesquisa metodológica, seguindo os passos de tradução, síntese, retrotradução e avaliação pelo comitê de especialistas. Participaram dois tradutores, dois retrotradutores e doze profissionais para o comitê de especialistas. Realizou-se o pré-teste com 10 pacientes com linfedema secundário ao câncer de mama. E o grau de concordância foi obtido pelo coeficiente de validade de conteúdo.

**Resultados:**

houve a necessidade de modificar 8 das 29 questões que compõem o questionário, as quais demonstraram discordância idiomática. Contudo, mesmo que tais alterações tenham sido realizadas, não houve indicação de prejuízos, já que foi alcançada a confiabilidade do conteúdo através da obtenção de 0,90 pelo coeficiente de validade.

**Considerações finais:**

o instrumento foi traduzido e adaptado transculturalmente para o Brasil com alto grau de concordância.

## INTRODUÇÃO

O câncer de mama é um dos principais problemas de saúde entre as mulheres devido à sua alta taxa de mortalidade e morbidade. Em cinco anos, a taxa de sobrevivência no câncer de mama metastático é inferior a 30%, mesmo com quimioterapia adjuvante. A mais recente estimativa realizada pela *International Agency for Research on Cancer* (IARC) em 2020 aponta que o câncer de mama feminino já ultrapassou o câncer do pulmão como a principal causa de incidência global de câncer, com uma estimativa de 2,3 milhões de novos casos, representando 11,7% de todos os casos de câncer^(1, 2)^.

Para o Brasil, a estimativa para cada ano do triênio 2023-2025 aponta que ocorrerão 704 mil casos novos de câncer (483 mil, excluindo os casos de câncer de pele não melanoma). Dentre estes, o mais incidente é o câncer de mama, correspondendo a 20,3 % dos casos de tumores em mulheres, corroborando com a tendência mundial^(3)^.

Os avanços no tratamento permitem uma gama de terapias, que podem incluir cirurgia, radioterapia, quimioterapia, hormonioterapia e terapia biológica (terapia alvo), dependendo da fase da doença (estadiamento) e tipo de tumor. Entre eles, a dissecção axilar cirúrgica e/ou radioterapia em região de gânglios linfáticos estão relacionadas ao surgimento de uma importante comorbidade, o linfedema^(4, 5, 6)^.

Por não haver padronização da avaliação do linfedema, sua incidência após a cirurgia de câncer da mama varia consideravelmente. Descreve-se uma incidência em torno de 9 a 40%, sendo 24 a 49% após a mastectomia, 4 a 28% após a tumorectomia com dissecção axilar e 5 a 34% após a cirurgia e radioterapia^(7)^.

O linfedema ocorre devido à obstrução ou ausência do fluxo linfático e é caracterizado pelo acúmulo extracelular de água, proteínas plasmáticas, células sanguíneas extravasculares e produtos celulares decorrentes deste transporte linfático deficiente, podendo estar associado a outras complicações como celulite, erisipela, linfangite e, muito raramente, ao linfangiossarcoma^(7, 8, 9, 10, 11)^. É uma condição crônica e progressiva, associada principalmente à linfadenectomia, radioterapia em cadeia de drenagem e obesidade. Embora essas alterações não tenham potencial letal, acabam causando diversos distúrbios no membro acometido, com repercussões estéticas, psicossociais e funcionais^(12, 13, 14)^.

A funcionalidade é o terceiro maior indicador em saúde, sendo uma ferramenta que auxilia na orientação terapêutica ao colocar o ambiente como facilitador ou dificultador no desempenho de funções e ações^(15, 16, 17, 18)^. O câncer de mama tem um impacto importante sobre a funcionalidade, mas indicadores funcionais não são tão estudados quanto os indicadores de sobrevida e mortalidade. Instituir avaliação de funcionalidade é uma tendência na agenda global de incorporação de desfechos e deve ser considerada ao avaliar o paciente com câncer, com e sem linfedema, uma vez que são conhecidos seus danos na esfera física, social e emocional^(19, 20, 21, 22)^. Importante destacar que as implicações negativas de barreiras no que tange ao acesso ao tratamento precoce também são possivelmente determinantes para a redução da funcionalidade em mulheres com câncer de mama^(23)^.

Diante da escassez de instrumentos traduzidos e validados para o português, capazes de auxiliar na avaliação da funcionalidade relacionada ao linfedema, especificamente pós cirurgia axilar para tratamento do câncer de mama, optamos pela tradução e adaptação transcultural do questionário *Lymphoedema Functioning, Disability and Health Questionnaire Lymphoedema* (LYMPH-ICF), que já apresenta validação em outras línguas.

O uso de escalas e questionários não específicos para linfedema de membros superiores limita a qualidade metodológica de estudos por não fornecer dados relevantes sobre a experiência dessa população. Além disso, ao ser elaborado com base na Classificação Internacional de Funcionalidade, Incapacidade e Saúde (CIF), o instrumento engloba diferentes aspectos relacionados às questões físicas, mentais, domésticas, sociais e de mobilidade, o que o torna uma importante ferramenta de avaliação na prática clínica daqueles que lidam com o linfedema, ao permitir a compreensão dos danos funcionais, das limitações impostas à realização de atividades diárias e da restrição de participação social.

## OBJETIVO

Realizar a tradução e adaptação transcultural do instrumento “*Lymphoedema Functioning, Disability and Health Questionnaire Lymphoedema* - LYMPH-ICF” para a língua portuguesa do Brasil.

## MÉTODOS

### Aspectos éticos

O desenho do estudo atendeu às normas contidas na Resolução do Conselho Nacional de Saúde nº 466/2012 e foi aprovado pelo Comitê de Pesquisa e Ética da Universidade Federal de São Paulo (UNIFESP), Certificado de Apresentação para Apreciação. Inicialmente, foi solicitada à Dra. Nele Devoogdt, primeira autora da versão original e da versão traduzida para o inglês, autorização por via eletrônica para a elaboração deste estudo. Todos os participantes assinaram o Termo de Consentimento Livre e Esclarecido.

### Tipo de estudo

Trata-se de um estudo metodológico de tradução e adaptação transcultural do instrumento “*Lymphoedema Functioning, Disability and Health Questionnaire Lymphoedema* - LYMPH-ICF” para a língua portuguesa do Brasil. O questionário LYMPH ICF, com embasamento terminológico da Classificação Internacional de Funcionalidade (CIF), avalia os prejuízos relacionados à funcionalidade, limitação de atividade e restrição de participação em pacientes pós cirurgia axilar decorrente do câncer de mama. Contendo 29 perguntas, foi construído com base nas informações de pacientes com linfedema secundário ao câncer de mama.

A pontuação das respostas é dada pelo paciente por meio de uma escala numérica de 11 pontos (0 a 10). Para cada pergunta, o paciente deverá indicar o número que melhor corresponde à sua situação. Será atribuído “0” se o paciente não apresentar nenhum problema relacionado à sua queixa, e “10” se o paciente apresentar problemas muito sérios com a queixa descrita. O item “não se aplica” simboliza que a queixa não se aplica ao paciente. As perguntas estão agrupadas em 5 blocos: 1) questões físicas: dor, sensação da pele e movimento (7 questões); 2) função mental (4 questões); 3) atividades domésticas (4 questões); 4) atividades de mobilidade (8 questões) e 5) atividades sociais e de vida (6 questões). Quanto maior a pontuação, pior o impacto do linfedema na funcionalidade da paciente com câncer de mama.

Os escores dos domínios variam de 0 a 100, em que o valor total é obtido pela soma das pontuações das 29 perguntas divididas pelo número total de respostas multiplicado por 10. Assim, o autor da versão original, em relação às deficiências da função, destaca que, de acordo com a taxonomia da Organização Mundial de Saúde (OMS), as limitações de atividades e restrições de participação são classificadas de: 0 a 4% (sem problemas), 5 a 24% (problema leve), 25 a 49% (problema moderado), 50 a 95% (problema grave) e 96 a 100% (problema muito grave)^(24)^.

### Procedimento metodológico

Para adaptar o LYMPH-ICF, foram seguidas as recomendações do Guideline de BEATON^(25)^, que visam proporcionar uma equivalência semântica, idiomática, cultural e conceitual entre o instrumento original e a versão adaptada. Deste modo, as cinco etapas necessárias para a Tradução e Adaptação Transcultural ( TAT ) de um instrumento foram realizadas, sendo elas a tradução, a síntese das traduções, a retrotradução, a avaliação do comitê de especialistas e o pré-teste, conforme apresentado na Figura 1.

**Figura 1:** Etapas de tradução e adaptação transcultural

Notas: T1/T2: síntese das traduções pelos autores; VB1: Primeira Versão Brasileira; VBF: Versão Brasileira Final.

### Cenário do estudo e coleta dos dados

O cenário do estudo e os dados obtidos foram distintos de acordo com cada etapa da pesquisa. Na primeira etapa, dois tradutores brasileiros independentes, com experiência na área de oncologia e linfologia e cientes do objetivo da pesquisa, realizaram a tradução inicial da versão em inglês para o português, denominados T1 e T2. Depois desta etapa, as traduções (T1 e T2) foram sintetizadas pelos pesquisadores responsáveis em uma versão única traduzida para o português (T1/2).

Na etapa de retrotradução (*back-translation*), a síntese (T1/2) foi encaminhada para dois tradutores nativos da língua inglesa para realizar a retrotradução na língua de origem do instrumento (BT1 e BT2). Esses autores desconheciam o objetivo da pesquisa. As versões do instrumento (a original, a síntese das traduções para o português e a retrotradução) passaram pela etapa de validação semântica e de conteúdo do instrumento através de um comitê de 12 especialistas de diversos estados brasileiros (São Paulo, Rio de Janeiro, Mato Grosso, Rio Grande do Sul, Brasília, Bahia e Santa Catarina) que foram selecionados por meio da consulta ao currículo na Plataforma Lattes, da média de tempo de experiência de 14,25 anos e a partir da observação dos critérios de inclusão subsequentes: nacionalidade brasileira, domínio da língua inglesa e expertise em pelo menos uma das seguintes áreas: linfologia, oncologia e/ou mastologia. O convite aos selecionados e as instruções processuais foram enviadas por meio eletrônico.

Em relação à tradução e adaptação transcultural, a literatura mostra-se extremamente divergente quanto ao número de participantes necessário para a formação do comitê de especialistas. Nenhum consenso aponta um único padrão de referência, mesmo que em torno de 30 diretrizes sejam identificadas. Portanto, os especialistas foram selecionados de acordo com os números apresentados em outros estudos como o referenciado^(26)^.

Após a comparação das versões com o texto original, possíveis falhas em 8 questões foram detectadas e conduzidas a análises em relação aos pontos discordantes. Ao minimizar os possíveis vieses linguísticos, culturais e de compreensão, viabilizou-se a construção da primeira versão brasileira do instrumento (VB1).

Em seguida, um pré-teste com a VB1 foi realizado para identificar se os procedimentos foram adequados ou se algum item continuou incompreensível. Essa etapa possibilitou a identificação e correção de possíveis erros antes da confecção da pesquisa. O pré-teste contou com a participação de 10 mulheres com linfedema, seguindo a versão publicada do TAT em relação ao número de participantes requerido para a elaboração do questionário para linfedema de membros inferiores. As pacientes indicadas foram recrutadas no ambulatório de Fisioterapia da Disciplina de Mastologia do Departamento de Ginecologia da Universidade Federal de São Paulo (UNIFESP). Elas apresentavam linfedema de membros superiores secundário ao câncer de mama, de qualquer grau, e possuíam escolaridade mínima de primeiro grau completo. Foram excluídos os pacientes não alfabetizados e com alterações visuais ou cognitivas, que dificultariam a leitura e compreensão do questionário.

O estudo também coletou amostras de variáveis demográficas (gênero, idade, estado civil) e clínicas (data do diagnóstico de linfedema e grau) com o objetivo de caracterizar a população estudada.

Após o pré-teste, foi iniciado o processo de elaboração da versão final, em que verificou-se o consenso entre o comitê de especialistas em relação à semântica (se foi feita a transferência de sentido dos conceitos presentes nas questões do instrumento original para a nova versão), as equivalências idiomáticas (se as expressões dos idiomas e os coloquialismos foram traduzidas corretamente), o fator cultural (se os itens do questionário procuram capturar a experiência da vida diária), e o conceitual (se foi realizada a análise dos conceitos e dos significados das palavras no contexto cultural inglês e brasileiro)^(27, 28)^.

### Análise dos dados

Finalizado o pré-teste, as questões foram novamente submetidas ao comitê de especialistas para avaliar a proporção de concordância semântica, idiomática, cultural e conceitual por meio do Coeficiente de Validade de Conteúdo (CVC). A avaliação das questões é feita por meio de uma escala tipo Likert de 4 pontos, em que 1 significa “não é claro”; 2 “pouco claro”; 3 “bastante claro”; e 4 “muito claro”. O resultado foi calculado somando-se as pontuações atribuídas aos itens 3 e 4 e pela divisão do resultado pelo número total de respostas. O valor superior a 0,80 é considerado satisfatório para assegurar o grau de concordância entre os especialistas.

## RESULTADOS

Depois que os procedimentos iniciais (tradução e retrotradução), demonstrados no Quadro 1, foram feitos para adaptar a LYMPH ICF adequadamente para o português do Brasil, foi realizada a avaliação do comitê de especialistas, que sugeriram pequenas alterações da versão T1/2 para o melhor entendimento das participantes, sendo acrescentados termos mais usuais do dia a dia da população brasileira.

**Quadro 1:** Questões versão original, tradução e retrotraduções das questões do LYMPH-ICF

Enunciado	Original	Tradução T1/T2	Retrotradução 1	Retrotradução 2
**Dor, sensações na pele, funções e movimento**				
	*Does your arm:*	Em relação ao seu braço:	*Does it arm:*	*Does it arm:*
1	*Feel heavy?*	Sente pesado?	*Feel heavy*	*Feel heavy?*
2	*feel stiff?*	Sente rígido?	*Feel stiff?*	*Does it feel tight?*
3	*Feel swollen?*	Sente inchado?	*Feel swollen?*	*Does it feel swollen?*
4	*Feel like it has lost strength*	Sente que perdeu a força?	*Feel as if you have lost your arm’s strength?*	*Do you feel like it has lose strength?*
5	*Tingle?*	Formigamento?	*Tingling?*	*Does it tingle?*
6	*Hurt?*	Dor?	*Any pain?*	*Does it hurt?*
7	*Have a tense skin?*	Tem a pele tensa?	*Have tense skin?*	*Is the skin tight?*
***Função Mental***				
8	*Due to you arm problems Do you feel sad?*	Devido ao problema no seu braço você se sente triste?	*Due to problems with your arm: Do you feel sad?*	*Do you feel sad?*
9	*Do you feel discouraged?*	Você se sente desencorajado?	*Do you feel discouraged?*	*Do you feel discouraged?*
10	*Do you have a lack of self-confidence?*	Você se sente falta de auto-confiança?	*Do you feel a lack of self-confidence?*	*Do you feel a lack of self-confidence?*
11	*Do you feel stressed?*	Você se sente estressado?	*Do you feel stressed?*	*Do you feel stressed?*
***Atividades domésticas***				
	*How well are you able to*	Quão bem você é capaz	*How well are you able to*	*How well are you able to*
12	*Clean? (scrub, vaccum, mop).*	Limpar? (esfregar, aspirar, passar pano)	*Cleaning? (rubbing, use a vacuum cleaner, wipe with a cloth)?*	*Clean?(scrubbing, vacuuming, dusting)?*
***Atividades domésticas***				
13	*Cook?*	Cozinhar?	*Cook?*	*Cooking?*
14	*Iron?*	Passar ferro?	*Iron clothes?*	*Ironing?*
15	*Work in the garden?*	Trabalhar no jardim?	*Work in the garden?*	*Work in the garden?*
**Atividades de mobilidade**				
	*How well are you able to*	Quão bem você é capaz de	*How well are you capable of*	*How well are you able to*
16	*Perform tasks with the arm elevated (eg, hang out the laundry)*	Executar tarefas com o braço elevado (por exemplo, estender a roupa)	*Performing tasks with a raised arm (such as putting clothes on the line)?*	*Perform tasks with your arm elevated (for instance, hanging clothes)?*
17	*Lift or carry heavy objects (eg, leg a filled bucket or shopping bags)?*	Levantar ou carregar objetos pesados (por exemplo, um balde cheio ou sacola de compras)?	*Lift or carry heavy objects (such as a bucketful of water or a full shopping bag)?*	*Lift or carry heavy objects (for instance, a filled bucket or a grocery bag)?*
18	*Sleep on the affected side?*	Dormir sobre o lado afetado?	*Sleeping on the affected side?*	*Sleep over the affected side?*
19	*Perform computer work?*	Fazer trabalho no computador?	*Using a computer?*	*Work on your computer?*
20	*Sunbathe*	Tomar sol?	*Sitting out in the sun?*	*Sunbathing?*
21	*Drive a car?*	Dirigir um carro?	*Driving a car?*	*Drive a car?*
22	*Walk (>2 km)?*	Andar (>2 km)?	*Walking (>2 km)?*	*Walk (>2 km)?*
23	*Ride a bike?*	Andar de bicicleta?	*Riding a bike?*	*Ride a bike?*
**Atividades sociais e de vida**				
	*How well are you able to*	Quão bem você é capaz de	*How well are you capable of*	*How well are you able*
24	*Go on vacation?*	Ir de férias?	*Going on a holiday?*	*Take a vacation?*
25	*Perform your hobbies?*	Realizar seus passatempos?	*Dedicating yourself to you hobbies?*	*Do your hobbies?*
26	*Practice sports?*	Praticar esportes?	*Engaging in sports activities?*	*Practice sports?*
27	*Wear your clothes of choice*	Usar suas roupas escolhidas?	*Using a given item of clothing?*	*Use chosen clothes?*
28	*Do your job?*	Fazer seu trabalho?	*Performing at your job?*	*Perform your job?*
29	*Do social activities (eg, going to parties, concerts, restaurant)?*	Fazer suas atividades sociais (por exemplo, ir a festas, concertos e restaurantes)?	*Conduct social activities (i.e.: go to parties, concerts or restaurants)?*	*Perform your social activities (for instance, go to parties, concerts, restaurants)?*

*Legenda: T1: versão traduzida 1, T2: versão traduzida 2.*

No enunciado, o termo “quão bem você consegue?” foi substituído por “você consegue?”, enquanto que mudanças mais discretas foram feitas nas 8 questões que tiveram discordância idiomática e conceitual, conforme apresentado no Quadro 2, que levou à primeira versão brasileira (VB1).

No pré-teste da VB1 foram incluídas 10 mulheres com média de idade de 55,6 anos (dp± 13,65), sendo 50% brancas. Das pacientes, 100% passaram por linfadenectomia axilar e radioterapia para tratamento do câncer de mama, desenvolvendo linfedema secundário ao tratamento oncológico. O linfedema havia sido diagnosticado há 20,6 meses (dp± 21,41), em média. Pelo menos uma paciente respondeu “não se aplica” em 8 das 29 perguntas do questionário. Ressalta-se que as questões foram marcadas como “não se aplica” por motivos exclusivamente da rotina da paciente, não por dificuldade de compreensão ou por não ser uma realidade da população brasileira de maneira geral. As alternativas que obtiveram “não se aplica” foram: questão 14: “passar ferro”; questão 15: “trabalhar no jardim”; questão 18: “dormir sobre o lado afetado”; questão 19: “fazer trabalho no computador”; questão 21: “dirigir um carro”; questão 22: “andar >2 km”; questão 23: “andar de bicicleta”; e questão 26: “praticar esportes”.

Após a avaliação das respostas do Comitê de Especialistas, que demonstrou grau de concordância pelo IVC de 0,90 (Figura 2), não foram necessárias alterações finais do instrumento traduzido, sendo considerado como a Versão Brasileira Final (VBF) adequada para ser aplicada na população estudada.

**Quadro 2:** Alterações realizadas após avaliação do comitê de especialistas e consenso entre os pesquisadores principais

**Questão**	**T1/T2**	**VB1**
**Enunciado**	Quão bem você é capaz?	Você consegue?
2	Rígido?	Endurecido?
6	Dor?	Dor no braço?
7	A pele tensa?	A pele esticada?
9	Desencorajada?	Desanimada?
16	Passar ferro?	Passar roupa?
19	Fazer trabalho no computador?	Trabalhar no computador?
24	Ir de férias?	Sair de férias?
27	Usar suas roupas escolhidas?	Usar as roupas que escolheu?

Legenda: T1/T2 Síntese das versões traduzidas, VB1: Primeira Versão Brasileira

**Figura 2:** Concordância do comitê de especialista em relação à versão brasileira final

Após a avaliação das respostas do Comitê de Especialistas, não foram necessárias alterações finais do instrumento traduzido, sendo considerado como a Versão Brasileira Final (VBF) adequada para ser aplicada na população estudada.

## DISCUSSÃO

Questionários de avaliação são ferramentas habituais nas diferentes áreas de saúde, muitas vezes utilizados em populações diferentes daquelas para as quais foram originalmente criados. Nestes casos, é necessária a adaptação transcultural na preparação e aplicação dos questionários, já que sua validade não depende somente de tradução, mas também de contextualização ao novo cenário cultural, para que mantenha suas propriedades psicométricas^(27, 29)^.

As traduções iniciais mostraram resultados próximos, e os tradutores não apontaram dificuldades, porque são brasileiros com experiência na área da linfologia e oncologia, então os desvios semânticos foram minimizados. Além disso, a atuação dos tradutores de forma independente garantiu que fossem evitados equívocos interpretativos e particularidades no modo de escrever. As traduções do título e das instruções de T1 e T2 do questionário LYMPH-ICF foram parecidas e não precisaram de mudanças quando transformadas em T1/T2.

Sendo obedecidos os critérios de BEATON (2000), este que sugere que a retrotradução deve ser cega (os retrotradutores não devem ter acesso ao questionário original) e que eles devem ser nativos na língua original do documento, fluentes na língua-alvo e não devem ter conhecimento técnico sobre o as-sunto^(25)^. A síntese das traduções passou por uma retrotradução (*back-translation*), cujo objetivo é identificar palavras que não ficaram claras na língua original e encontrar inconsistências ou erros conceituais na versão brasileira final, quando comparada com a versão original^(30)^.

Foi identificada a necessidade de realizar pequenas alterações no processo de retrotradução após a avaliação do comitê de especialistas, pois os retrotradutores utilizaram pronomes e preposições diferentes, bem como palavras sinônimas, o que culminou em diferentes retrotraduções com possível discordância em relação a equivalência idiomática em 8 questões. Apesar de terem sido realizadas pequenas mudanças como sugerido em alguns itens, elas não alteraram a dimensão teórica do instrumento se comparada à original.

O pré-teste é uma aplicação prévia do instrumento em uma pequena amostra que reflete as características da população alvo, a fim de avaliar a adequação dos itens em relação ao seu significado e à sua dificuldade de compreensão^(31)^. Devido à baixa escolaridade da população atendida em nosso serviço, essa etapa demandou uma explicação sobre a maneira de fazer a pontuação das dificuldades através da escala analógica visual usada no questionário, mas isso não desabona a aplicabilidade do questionário na prática clínica, visto que a clareza da linguagem teórica das questões do instrumento foi confirmada pelo alto nível de concordância dos especialistas, com valor CVC = 0,90 na escala de Likert, como preconizado na literatura.

Em recente revisão sistemática para avaliar as propriedades psicométricas dos questionários que medem a qualidade de vida de pacientes com linfedema secundário ao câncer de mama, dentre os 9 instrumentos avaliados usando o checklist COSMIN Risk of Bias, o LYMPH ICF foi classificado como o que apresenta a melhor evidência de validade de conteúdo em relação a relevância, abrangência e compreensibilidade^(31)^.

Em termos de aspectos de viabilidade, a LYMPH ICF tem perguntas curtas, claras e diretas, e uma escala numérica de 11 pontos que pode ser facilmente compreendida pelos pacientes e pelos clínicos. O questionário também vem com um cálculo de pontuação fácil e leva de 5 a 10 minutos para ser concluído^(31)^.

Os procedimentos empregados para adaptar e traduzir um instrumento para uma nova língua consistem em etapas distintas, que requerem atuação com impessoalidade, uniformidade e obediência ao segmento metodológico proposto, de forma que os valores refletidos pelo instrumento e os significados de seus itens se mantenham equivalentes entre as culturas para que a sua tradução tenha a mesma aplicabilidade do original^(32, 33)^.

Os resultados encontrados estão de acordo com outros descritos na literatura científica, o que torna a possibilidade de uma versão em português real, visto que o instrumento original^(27)^foi submetido a tradução para diversos idiomas como o francês, o dinamarquês e o chinês, através da utilização de etapas semelhantes, além da avaliação de questões psicométricas que garantem a confiabilidade e validade do LYMPH-ICF e da versão semelhante para linfedema de membros inferiores^(34, 35, 36, 37)^.

### Limitações do estudo

Embora o estudo tenha seguido rigorosos procedimentos metodológicos, algumas limitações devem ser consideradas. Em primeiro lugar, a amostra utilizada no pré-teste foi relativamente pequena, composta por apenas 10 mulheres com linfedema secundário ao câncer de mama. Uma amostra mais ampla poderia proporcionar uma avaliação mais abrangente da compreensão e aplicabilidade do questionário, possibilitando uma validação mais robusta da versão brasileira. Além disso, a seleção dos participantes se restringiu a um único centro de saúde, o que pode limitar a generalização dos resultados para outras populações ou contextos de atendimento médico.

Outra limitação potencial está relacionada à homogeneidade da amostra em termos de características sociodemográficas, como idade e nível de escolaridade. A maioria das participantes do estudo apresentava uma média de idade em torno de 55 anos e possuía escolaridade mínima de primeiro grau completo. Essa homogeneidade pode afetar a representatividade dos resultados, especialmente quando consideramos pacientes com diferentes faixas etárias ou níveis educacionais. Uma abordagem mais diversificada poderia proporcionar insights adicionais sobre a compreensão e aceitação do instrumento em diferentes grupos populacionais, permitindo uma adaptação mais abrangente e inclusiva do questionário para o contexto brasileiro.

### Contribuições para a área da enfermagem, saúde ou política pública

Este estudo ofereceu um valioso instrumento para a área da enfermagem, saúde e política pública ao fornecer um questionário sobre funcionalidade, incapacidade e saúde do linfedema, traduzido e adaptado transculturalmente para o português do Brasil. Essa iniciativa permitirá aos profissionais avaliar e monitorar a presença e a progressão do linfedema em mulheres submetidas à cirurgia para tratamento do câncer de mama. Este é o primeiro passo crucial para o processo de validação do LYMPH-ICF, fornecendo uma ferramenta vital para a prática clínica e a pesquisa na área, além de contribuir significativamente para a melhoria dos cuidados prestados a essas pacientes.

## CONSIDERAÇÕES FINAIS

Após o processo de tradução e adaptação transcultural, os resultados obtidos na avaliação da semântica, das equivalências idiomáticas e dos fatores culturais e conceituais possibilitaram a criação de uma versão do instrumento em português do Brasil com um alto grau de concordância (CVC 0,90). Recomenda-se, a partir deste ponto, sua aplicação em estudos futuros com a população brasileira que sofre de linfedema secundário ao câncer de mama, visando à análise psicométrica, confiabilidade estatística e validação do instrumento.

## Data Availability

https://doi.org/10.48331/scielodata.2IQUF1
